# Ectopic pancreas appearing as a giant gastric cyst mimicking gastric lymphangioma: a case report and a brief review

**DOI:** 10.1186/s12876-021-01686-9

**Published:** 2021-04-06

**Authors:** Xuemei Liu, Xinglong Wu, Biguang Tuo, Huichao Wu

**Affiliations:** 1 Department of Gastroenterology, Affiliated Hospital of Zunyi Medical University, Zunyi, 563003 Guizhou Province China; 2 Department of Pathology, Affiliated Hospital of Zunyi Medical University, Zunyi, 563003 Guizhou Province China

**Keywords:** Ectopic pancreas, Serous oligocystic adenoma, Giant gastric cyst, Differential diagnostics and treatment, Gastric lymphangioma, Rare case

## Abstract

**Background:**

Ectopic pancreas (EP) is defined as pancreatic tissue that lacks anatomical or vascular communication with the normal body of the pancreas. Despite improvements in diagnostic endoscopy and imaging studies, differentiating ectopic pancreatic tissue from gastric submucosal diseases remains a challenge.

**Case presentation:**

Here, we present a case of a 44-year-old woman with severe epigastric pain. Initially, gastric lymphangioma was highly suspected due to a well-demarcated protruding mass with a large size that occurred in the submucosal layer of the gastric antrum and appeared as a cystic lesion. The final correct diagnosis of gastric EP was made during surgery.

**Conclusion:**

Gastric EP with serous oligocystic adenoma appearing as a giant gastric cyst is extremely rare. The difficulty of making an accurate diagnosis and differential diagnosis is highlighted, which may provide additional clinical experience for the diagnosis of EP with serous oligocystic adenoma in the stomach.

## Background

Ectopic pancreas (EP) refers to healthy pancreatic tissue that lacks anatomical, vascular or neural communication with the normal pancreas; EP was probably first described in the eighteenth century when it was found in an ileal diverticulum [1]. EP can be detected in the gastrointestinal (GI) tract, biliary system, liver, lung, mediastinum, and brain [1–4]. In the GI tract, the most common location for its presence is the stomach, followed by the duodenum (25–35%) and jejunum (16%) [1–4]. EP is usually found incidentally and is asymptomatic; however, it may become symptomatic when complicated by inflammation, bleeding, obstruction or malignant transformation [3–6]. Generally, it is difficult to make the correct pathological diagnosis from a typical endoscopic mucosal biopsy to distinguish EP from other gastric submucosal diseases. Herein, we report the case of a 44-year-old female with ectopic pancreas and serous oligocystic adenoma in the gastric antrum that mimicked a gastric lymphangioma.

## Case presentation

**Fig. 1:**
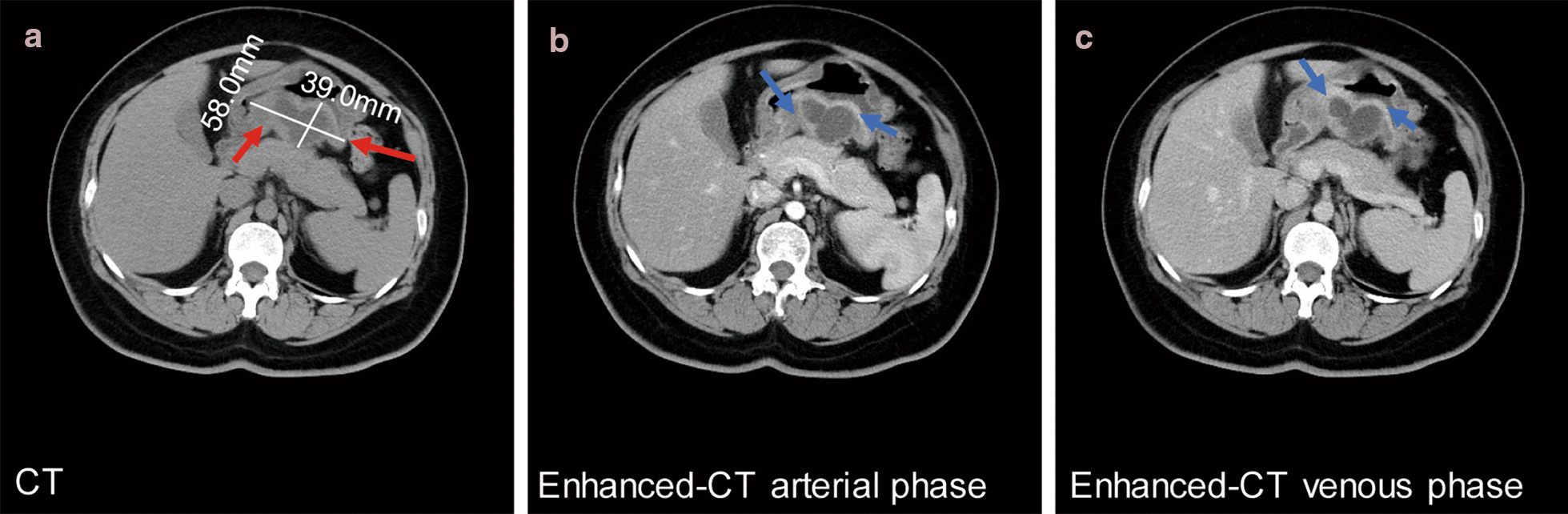
A heterogeneous cystic and solid submucosal tumor in the stomach was detected by CT. **a** The size and location of the tumor are shown on CT by the red arrows. **b**, **c** The enhanced CT scans in the arterial phase and venous phase, respectively, and blue arrows indicate thick cystic walls

**Fig. 2:**
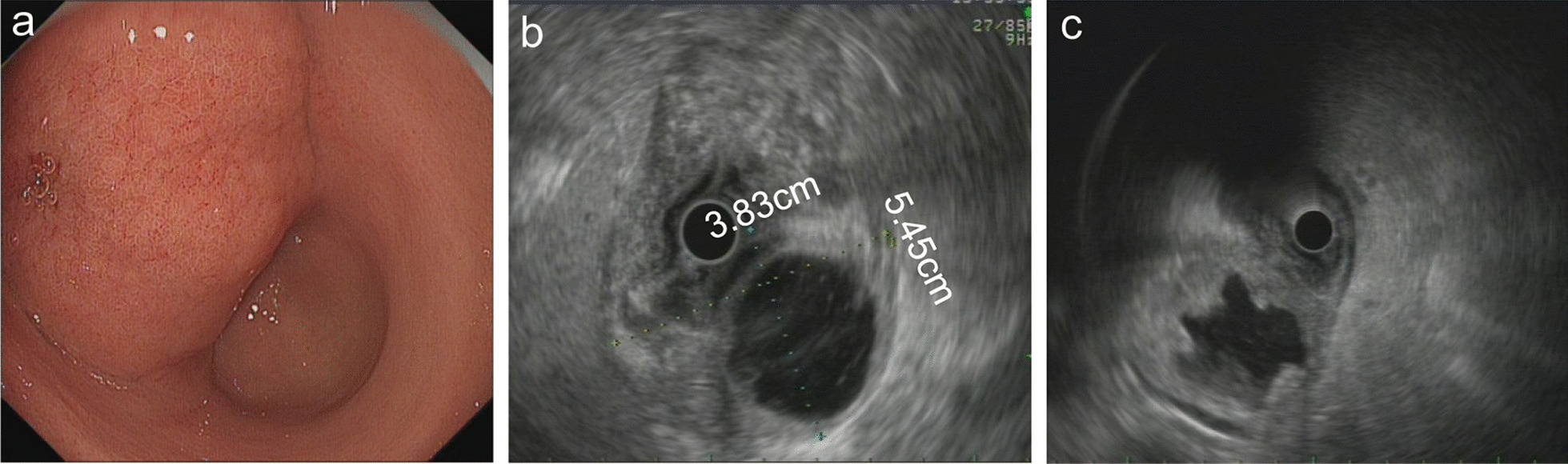
Esophagogastroduodenoscopy and EUS images of EP. **a** Esophagogastroduodenoscopy showed a lesion elevated from the submucosa that was located from the front wall of the gastric antrum to the gastric angle with normal overlying mucosa. **b** EUS noted a smooth 5.5 * 3.8 cm cystic and solid anechoic lesion in the submucosal layer, and the cystic wall became thicker when approaching the pylorus (**c**)

A 44-year-old female was admitted to our hospital due to epigastric pain with a duration of 6 months. There was no obvious cause of the paroxysmal dull abdominal pain when the pain was intense, radiating to the back. There was no history of loss of appetite, postprandial vomiting or gastrointestinal bleeding. Proton pump inhibitors were started but did not relieve the symptoms. Physical examination and her routine laboratory investigations were unremarkable. Computed tomography (CT) identified a 5.8 * 3.9 cm epigastric mass that appeared to be a heterogeneous cystic and solid submucosal tumor arising from the thick posterior wall of the antrum proximal to the pylorus (Fig. 1a). Enhanced CT displayed irregular mixed cystic and solid lesions with thick walls and intracystic fluid. In the arterial phase, the solid part was unevenly enhanced, and the thick wall of cystic cavity was obviously enhanced until the venous phase (Fig. 1b, c). Esophagogastroduodenoscopy showed a lesion elevated from the submucosa that was located from the posterior wall of the gastric antrum to the gastric angle with normal overlying mucosa (Fig. 2a). Endoscopic ultrasound (EUS) noted a smooth 5.5 cm + 3.8 cm mixed cystic and solid anechoic lesion in the submucosal layer, partially in the muscularis propria and serosa (Fig. 2b); the cystic wall became thicker when approaching the pylorus (Fig. 2c), with features suggestive of lymphangioma. Because of the size, the unknown pathology, and its involvement of the antrum whereby an attempt at local resection would have markedly narrowed the antrum, a decision was made to perform a distal gastrectomy. Clear transparent fluid was found in this cystic mass when opened in the pathology laboratory.

**Fig. 3:**
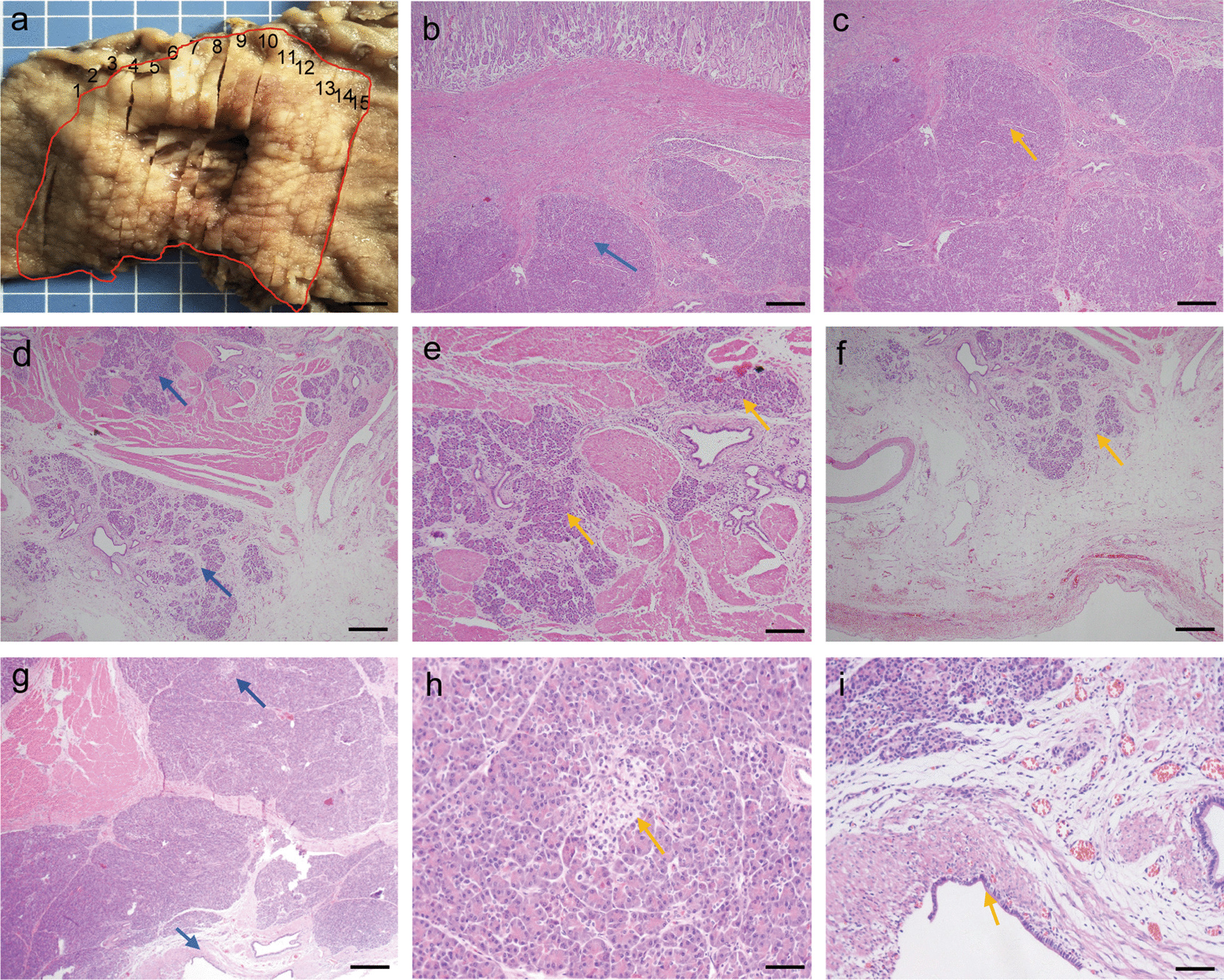
Histology of the surgical specimen. **a** Image of the surgical specimen. Numerous acini (**b**), few ducts (**d**) and islet cells (**h**) were found in the submucosa, muscularis propria and serosa. Cysts were lined by a single layer of short columnar or cubic serous epithelia (**g**). **c** Is a higher magnification image of **b**; **e**, **f** are higher magnification images of **d**; **h**, **i** are higher magnification images of **g**. **b**, **d** Represent slide 9 in **a**, and **g** is slide 5 in **a**. Blue arrows indicate EP tissues, and yellow arrows indicate higher magnification lesions. Scale bars represent 200 μm (**a**, **b**, **d**, **g**), 100 μm (**c**, **e**, **f**), and 50 μm (**h**, **i**)

Histopathologic examination of the resected mass showed aberrant pancreatic tissues, including numerous acini, few ducts and islet cells involving the submucosa, muscularis propria and serosa; furthermore, the cysts were lined with a single layer of short columnar or cubic serous epithelia with clear cytoplasm, and all of these histological features supported a final diagnosis of EP with serous oligocystic adenoma (SOA) (Fig. 3a–i). The postoperative course was uneventful, and she has been asymptomatic with no recurrence for 18 months to date.

## Discussion and conclusions

EP is defined as pancreatic tissue that lacks anatomical or vascular communication with the normal body of the pancreas [7, 8]. Generally, the most common heterotopic site is the stomach, including the antrum and prepyloric region on the greater curvature or posterior wall; EP is usually found incidentally and is asymptomatic. However, it may become symptomatic when complicated by inflammation, bleeding, obstruction or malignant transformation [3–5]. Symptoms are dependent upon the anatomical location and the size of the lesion. Generally, lesions greater than 1.5 cm in diameter are more likely to cause symptoms [9], and pain is one of the most common symptoms [10]. In this case, a middle-aged woman with epigastric pain, a 5.8 * 3.9 cm cystic and solid lesion with a thick gastric wall was detected by CT and EUS. One possible explanation for the pain might be associated inflammation of the involved tissue as reported [10], but we didn’t have the direct evident to have any inflammatory sign. Therefore, reason of the pain was unknown.

A previous report indicated that EP often localizes in the submucosa, muscularis mucosa, and serosa, with distributions of 73%, 17% and 10%, respectively [1], sometimes involving through all of these layers [1]. Generally, we can make a clinical diagnosis of EP with a size less than 2 cm by upper endoscopy and EUS. However, for a giant EP, although imaging studies such as CT, radiographic contrast studies, EUS, and upper endoscopy are of assistance in the initial assessment of patients, it is still difficult to make a correct pathological diagnosis from endoscopic biopsy to distinguish EP from other gastric submucosal diseases [1, 10], which is the reason for operative treatment [10]. The final correct diagnosis is made from the pathological diagnosis of surgical specimens, and four types of pathological classifications of EP have been identified by the groups of Heinrich and Gaspar-Fuentes: type I) typical pancreatic tissue with ducts, acini and islet cells; type II) numerous acini, few ducts, and no islet cells; type III) numerous ducts, few to no acini and no islet cells [11]; and type IV) endocrine islets without exocrine pancreatic tissue [12].

In this case, due to the large size and location of the lesion in the submucosa, the difficulty of endoscopic treatment, as well as the needs of patient, distal gastrectomy and Billroth II reconstruction were performed by laparoscopy. Histological analysis showed that numerous acini, few ducts and some endocrine islets were found in the submucosa, muscularis propria and serosa; thus, a type I lesion was considered according to the Heinrich and Gaspar-Fuentes classification. Previous studies have reported that cystic changes can be seen in EP tissue and that retention cysts are usually small (less than 1–2 cm in size) and lined by a single layer of normal pancreatic epithelium, whereas pseudocysts are usually larger with walls that lack an epithelial lining [13, 14]. Furthermore, pancreatic cysts are classified as neoplastic or nonneoplastic. Neoplastic cysts include serous cystadenomas (SCA), solid-pseudopapillary neoplasm (SPN), mucinous cystic neoplasm (MCN), intraductal papillary mucinous neoplasms (IPMN), and cystic pancreatic neuroendocrine tumors. Nonneoplastic cysts include simple cysts, lymphoepithelial cysts, and mucinous nonneoplastic cysts [15, 16]. In detail, serous cystic neoplasms (SCN) include SCA, VHL-associated serous cystic neoplasm, serous cystadenocarcinoma, and cystic neuroendocrine neoplasms [17]. SCA of the pancreas are uncommon benign neoplasms, which are composed of epithelial cells that produce serous fluid and show evidence of ductular differentiation, which are subdivided into serous micro cystic (glycogen-rich), and serous oligocystic adenoma (SOA) [17, 18]. SOA is defined as cystic epithelial neoplasms composed of ductular-type epithelial cells that produce a clear watery fluid, not contained mucinous, bilious and hemorrhagic. Morphologically, SOA is over 2 cm in size, and anechoic lesion was detected by EUS [18]. Gross pathology often demonstrates a giant cyst filled with serous fluid, by histology, the cysts are lined by a single layer of cuboidal or flattened epithelial cells with clear cytoplasm, and the periodic acid-Schiff (PAS) stain is positive owing to their intracytoplasmic glycogen. In our case, pathological analysis demonstrated that the cyst was lined by a single layer of short columnar or cubic serous epithelia with clear cytoplasm, as well as numerous acini and few ducts in the submucosa, muscularis propria and serosa, which strongly supported a final diagnosis of EP with SOA. EP with SOA appearing as a giant gastric cyst is relatively rare, providing a novel and atypical case for EP.

The main differential diagnoses with EP include gastrointestinal stromal tumors, gastrointestinal autonomic nerve tumors, gastric carcinoids, lymphomas and gastric carcinomas [3, 19, 20]. Moreover, gastric cystic lesions needed to be distinguished from different diseases, including gastric diverticula, gastric duplication, and gastritis profunda cystica. However, initially, gastric lymphangiomas have been highly suspected before surgery for the following reasons: most lymphangiomas manifest as cystic or cavernous lesions [19]. In our case, a well-demarcated protruding mass with a large size occurred in the submucosal layer of the gastric antrum and appeared as a cystic lesion, and except for severe epigastric discomfort, there were no abnormal laboratory findings or clinical signs, which may be explained as infection in cystic lesions because enhanced CT displayed irregular cystic and solid lesions with thick walls and flocculent contents, and the cystic wall was obviously thicker and enhanced from the arterial to the venous phase.

Additionally, gastric lymphomas can present with pain [21–23]. Endoscopically, lymphangioma presents as a submucosal tumor with normal overlying mucosa [23]. EUS images showing lesions composed of anechoic and lobulated structures located predominantly in the submucosa are also characteristic of gastric lymphangioma [22–26], but these lesions have very thin walls and little stromal or thickened wall Therefore, it is necessary to clarify gastric lymphangioma in the differential diagnosis of EP in clinical work.

In summary, gastric EP with serous oligocystic adenoma appearing as a giant gastric cyst is extremely rare. Despite improvements in diagnostic endoscopy and imaging studies, it remains a challenge to differentiate ectopic pancreatic tissue from gastric submucosal diseases, such as gastric lymphangioma. This study may provide additional clinical experience for the diagnosis of EP in the stomach.

## Data Availability

All information about the patient come from the Affiliated Hospital of Zunyi Medical University. The data used and analyzed during the current study are included in this article.

## Abbreviations

CT: Computed tomography
EP: Ectopic pancreases
EUS: Endoscopic ultrasound
IPMN: Intraductal papillary mucinous neoplasms
MCN: Mucinous cystic neoplasm
PAS: Periodic acid-Schiff
SCA: Serous cystadenomas
SCN: Serous cystic neoplasms
SOA: Serous oligocystic adenoma
SPN: Solid-pseudopapillary neoplasm
